# Some Points on the Modern Treatment of Syphilis

**Published:** 1909-03-27

**Authors:** 


### Surgery.

### SOME POINTS ON THE MODERN TREATMENT OF SYPHILIS.

Until comparatively recent times the treatment of a case of syphilis was regarded as a matter of routine. The stereotyped remedy was the administration of mercury by the mouth in the form of the pil. hydrarg., and the patient was condemned to take this daily for a prescribed period, usually two years. It is hardly necessary to say that the constant absorption of mercury led to distressing effects, the most troublesome of which were the colicky abdominal pain, associated with diarrhœa, and the stomatitis, which were observed to follow upon the administration of the drug. As a result patients in many cases desisted from the treatment, and, finding often that no symptoms followed this immediately, gave up the treatment altogether and were consequently never cured.

Other methods of administering mercury were therefore devised, and on the Continent they have to some degree supplanted the older and less satisfactory form of treatment. In this country we are more conservative, and the treatment of syphilis by the mouth is still largely practised.

In France, particularly at Aix-la-Chapelle, the administration of mercury by inunction is extensively employed, and the medical men there who have a large experience of it are well satisfied with the method. One of them, Dr. Lieven, recently read a paper before the Laryngological Section of the Royal Society of Medicine, in which he explained the tchnique.

Four to five grammes of grey ointment of the strength of 33 per cent. is evenly rubbed into his skin by the patient for twenty minutes daily, a different portion of skin being chosen on each occasion. This is continued for five days, during which the body is not washed with soap and the underclothes are not changed. On the sixth day the patient is thoroughly washed and then the process is repeated for another five days, and so on. At Aix the process of absorption is facilitated by giving a sulphur bath at 95° F. before each rubbing. The mercury, given in this way, is rubbed into the pores and converted into an albuminate, in which form it circulates.

The complications which commonly follow on pill treatment are not rare in the case of inunction, but they are not so distressing when they do occur, and are easily treated symptomatically.

As Dr. Lieven said, it cannot be denied that the inunction treatment has several obvious drawbacks. For instance, it is not easy for a patient to carry it out for himself in his own home, and less easy to conceal the fact that he is doing so. Also the method has been described as being unpleasant and unclean; but it is only fair to say, in extenuation of inunction, that, as practised at Aix, it leaves little to be desired, in spite of its disadvantages. Moreover, it is efficient withal, and relapses are not common. The latest treatment is the intramuscular injection of some mercury salt. Considering the rapidity with which this method has come to the front in the last few years, it is curious to reflect that it was first advocated and practised as long ago as 1867. Yet this is the case.

This method has obvious advantages over the others. It is the most reliable way of administering mercury. The unpleasant complications associated with pill treatment are quite uncommon, though, of course they can occur. It has this great advantage over the inunction method as practised at Aix—that it takes up no time and does not interfere with the patient's daily life, nor prevent him from following his ordinary occupation, an injection being given not more often than once a week in the case of insoluble solutions.

A great variety of solutions of the salts of mercury have been advocated from time to time as being the best to use. The truth of the matter is that we have not yet had sufficient experience of their different effects to be able to estimate their respective value.

The solutions are divided into two classes—the soluble and the insoluble. The soluble cause less pain, but are less reliable in their action, and have to be repeated at more frequent intervals than the insoluble—namely, every two days. Of the soluble preparations, the succinamide, the benzoate, and the sozoiodolate of mercury are all being extensively tried. Of the latter, a third of a grain may be given suspended in ten minims of water.

The various insoluble preparations of mercury which are in general use are (1) calomel suspended in sterilised olive oil; (2) grey oil, which consists of hydrarg. pur. ʒj.; adeps lanæ anhydr. ʒiv.; paraffin. liq. (carbolisat. ad 2 per cent.) ad ʒx.; and (3) the salicylate of mercury, which can also be suspended in sterilised olive oil or in vasenol. These themselves vary in their effects. Calomel is said to be the most effective in that it most quickly gets a case of malignant syphilis under control; but it also causes deep-seated pain at the site of injection, which may be very severe and last for a considerable time. This can be mitigated by the simultaneous introduction of stovaine. A further disadvantage is that intra-muscular abscesses have been observed to follow its use more frequently than the other insoluble salts. It does not therefore commend itself for routine work. For this the salicylate, which seems really to be half-way between the soluble and insoluble salts in its effects, is the best.

Recently experiments have been undertaken with a view to determining whether the injection of large doses of arsenic, in the form of an amyl-arsenate, has any therapeutic value in syphilis. Atoxyl was first given, but was discarded on account of its toxic properties, several cases of complete blindness being recorded. The drugs which are now being used are soamin in this country, and arsacetin in Germany. The various accounts are most conflicting, but this may be said with certainty: that, so far, there is no evidence to show that either of these preparations is as good as mercury in the treatment of syphilis.

